# Acylated Ghrelin is Protective Against 6-OHDA-induced Neurotoxicity by Regulating Autophagic Flux

**DOI:** 10.3389/fphar.2020.586302

**Published:** 2021-01-27

**Authors:** Xin He, Wei Yuan, Fei Liu, Juan Feng, Yanxia Guo

**Affiliations:** ^1^ Department of Neurology, Shengjing Hospital of China Medical University, Shenyang, China; ^2^ Department of Orthopedics, The First Hospital of China Medical University, Shenyang, China

**Keywords:** parkinson’s disease, ghrelin, autophagic flux, TFEB, lysosome

## Abstract

Parkinson’s disease (PD) is one of the most common neurodegenerative disorders, and our previous study revealed that autophagic flux dysfunction contributes to the neuron death in 6-OHDA-induced PD models. Acylated ghrelin is a neuropeptide that has a variety of actions in the central nervous system. In the current study, we aimed to investigate whether ghrelin is neuroprotective in 6-OHDA-induced rat model and SH-SY5Y cell model and whether it is related to autophagic flux regulation. We observed that ghrelin could effectively reduce apomorphine-induced contralateral rotation in 6-OHDA-induced PD rats, preserve the expression of tyrosine hydroxylase (TH) and increase the cell viability. It could upregulate the expression of autophagy related proteins like Atg7 and LC3-II and downregulate p62, and downregulate apoptosis related proteins like bax and cleaved caspase 3. SH-SY5Y cells transfected with adenovirus Ad-mCherry-GFP-LC3B further revealed that ghrelin could relieve the autophagic flux dysfunction induced by 6-OHDA. Lysotracker staining showed that ghrelin could reverse the decrease in lysosomes induced by 6-OHDA and immunofluorescence staining revealed a reverse of TFEB level in SH-SY5Y cells. Blocking autophagy activation with 3-methyladenine (3-MA) in rats treated with ghrelin and 6-OHDA showed no notable change in apoptosis-related markers, while blocking autophagosome fusion with lysosomes with chloroquine could notably reverse the downregulation of bax/bcl-2 ratio and cleaved caspase three expression by ghrelin. Additionally, knockdown ATG7, the upstream regulator of autophagy, with siRNA could further decrease the number of apoptotic cells in SH-SY5Y cells exposed to 6-OHDA and treated with ghrelin, while knockdown TFEB, a key transcription factor for lysosome biosynthesis and function, with siRNA could completely abolish the anti-apoptosis effect of ghrelin. These data suggest that ghrelin is neuroprotective in 6-OHDA-induced PD models via improving autophagic flux dysfunction and restoration of TFEB level.

## Introduction

Parkinson’s disease (PD) is one of the most common neurodegenerative disorders characterized by progressive loss of dopaminergic neurons in the substantia nigra (SN). It is characterized with motor dysfunction, mainly including symptoms of rigidity, tremor and postural instability. Current PD treatments only replace or boost existing dopamine, providing short-term symptomatic relief (Lim and Zhang, 2013). A curative therapy has yet to be developed as the pathogenesis of PD remains unrevealed. However, increasing evidence suggests that aberrant intracellular degradation systems are actively involved in PD progression (Senkevich and Gan-Or, 2019). Genetic mutations related to either ubiquitin signaling pathway or autophagy-lysosome pathway have been revealed to be important genetic causes or risk factors for PD (Walden and Muqit, 2017; Karimi-Moghadam et al., 2018).

Autophagy is a highly conserved cellular process that targets the long-lived proteins and damaged organelles for clearance and recycle. Macroautophagy (hereafter referred to as autophagy) is the most well studied type of autophagy, which is featured with the formation of a double-membrane vesicle that encloses targeted materials and further fuses with lysosomes for degradation. The rate of protein degradation through autophagy is termed autophagic flux, which is varied depending on the cell and tissue types (Klionsky et al., 2016). Increased autophagic flux and enhanced expression of autophagy markers have been reported in the carriers of G2019S-LRRK2-mutation, a familial form of PD (Su et al., 2015; Smith et al., 2016). Moreover, the endogenous G2019S-LRRK2 could induce notable changes in lysosome morphology and acidification and cause accumulation of insoluble α-synuclein and enhanced α-synuclein release (Schapansky et al., 2018). Our previous study on 6-OHDA-induced PD model has also revealed that autophagic flux dysfunction contributes to neuronal death, and either inhibiting excessive autophagosome formation or restoring lysosome function could confer certain neuroprotection (He et al., 2018), suggesting modulation of autophagic flux as a feasible therapeutic target for PD.

Ghrelin is a gut-derived neuropeptide firstly identified as a natural ligand of the growth hormone secretagogue receptor 1a (GHS-R1a). There are two forms of ghrelin in the circulation, namely, acylated ghrelin and unacylated ghrelin. Acylated ghrelin takes up 10% of total ghrelin, which can bind to the GHS-R1a located in many brain regions raging from the hypothalamus to the substantia nigra and olfactory bulb; while unacylated ghrelin exerts the physiological function through a receptor yet to be defined (Mani et al., 2014). Ghrelin could mediate various physiological functions, including release of hormones like growth hormone, regulation of gastrointestinal motility, stimulation of food intake, etc. (Yin and Zhang, 2016). Clinical evidence suggests that both total ghrelin and acylated ghrelin levels are decreased in PD (Song et al., 2017), suggesting a possible link between endogenous ghrelin level and the pathogenesis of PD.

In the current study, we mainly evaluated the action of acylated ghrelin (hereafter referred to as ghrelin) in the 6-OHDA-induced rat and SH-SY5Y cell models. We found that ghrelin could improve the motor deficits and cell viability, which was related to its receptor activation and amelioration of autophagic flux dysfunction and apoptosis inhibition. Despite ghrelin could upregulate autophagy induction, the restoration of TFEB expression and TFEB-mediated lysosome biogenesis play a more prominent role in its neuroprotection. Our findings suggest that ghrelin could be a promising agent in the treatment of PD.

## Material and Methods

### Chemicals and Antibodies

6-OHDA (#H116), apomorphine (#A4393), 3-methyladenine (3-MA, #M9281), chloroquine (CQ, #CC6628) and mouse anti-TH (#1299) monoclonal antibody were purchased from Sigma-Aldrich (St. Louis, MO, USA). [D-Lys3]-GHRP-6 (#1922/5) was purchased from R&D (Minneapolis, MN, USA). Antibodies against LC3B (#2775S) and cleaved caspase 3 (#9661) were obtained from Cell Signaling Technology (Beverly, MA, USA). The antibody against TFEB (A303-673A) was obtained from Bethyl Laboratories (Montgomery, TX, USA). Antibodies against p62 (#18420-1-AP), Bax (#50599-2-Ig) and Bcl-2 (#12789-1-AP) were obtained from Proteintech Group, Inc. (Chicago, IL, USA). Anti-GAPDH (#Ab103-01) antibody was purchased from Vazyme Biotech Co. (Nanjing, China). The HRP-conjugated anti-mouse (#ZB-2305) and anti-rabbit (#ZB-2301) antibodies were obtained from ZSGB-BIO (Beijing, China). DMEM/F12 culture medium, fetal bovine serum, penicillin/streptomycin solution and trypsin EDTA were obtained from Biological Industries (Beit Haemek, Israel).

### Animals

Male Sprague-Dawley rats weighing 200–220 g were purchased from Beijing HFK Bioscience Cooperation, China. All animal care and procedures were approved by Shengjing Hospital Medical Ethics Committee (2019PS054K(X1)). They were housed and bred in accordance with NIH Guidelines for the care and use of laboratory animals. The rats were housed in a12 h light/dark cycle schedule and with ad libitum access to food and water. For 6-OHDA-induced rat model, the animals were randomly divided into the following groups: 1) control group: rats received stereotaxic injection of normal saline at right striatum as described before (He et al., 2018); 2) ghrelin solely-treated group: rats received ghrelin injection (i.c.v., 100 ng); 3) 6-OHDA solely-treated group: rats received stereotaxic injection of 6-OHDA at right striatum as described before (He et al., 2018); 4) ghrelin+6-OHDA treatment group: rats received different doses of ghrelin (100, 200 and 400 ng/rat, i.c.v.) 30 min before intrastriatal injection of 6-OHDA, and to observe the long-term effect of ghrelin on 6-OHDA-induced dopaminergic neuron loss, rats received different doses of ghrelin for consecutive 14 days; 5) ghrelin + 6-OHDA+ [D-Lys3]-GHRP-6 group: rats received 100 ng ghrelin (i.c.v.) and intrastriatal injection of 10 μg [D-Lys3]-GHRP-6 30 min before intrastriatal injection of 6-OHDA as described before (Wei et al., 2015); 6) ghrelin + 6-OHDA + 3-MA group: rats received 100 ng ghrelin (i.c.v.) and intrastriatal injection of 200 nmol 3-MA 30 min before intrastriatal injection of 6-OHDA as described before (He et al., 2017); 7) ghrelin + 6-OHDA + CQ group: rats received 100 ng ghrelin (i.c.v.) and 10 mg/kg CQ (intraperitoneal injection, i.p.) 30 min before intrastriatal injection of 6-OHDA as described before (He et al., 2018). To study the effect of ghrelin on autophagy-lysosome pathway, the rats were sacrificed 1 day after corresponding treatments, as autophagy-related markers showed the most notable changes within 1 day in the 6-OHDA-induced rat model based on our previous findings (He et al., 2018). And to observe the long-term effect of ghrelin on rat behavioral changes and the survival dopaminergic neurons, the rats were sacrificed 5 weeks after corresponding treatments.

### Behavioral Testing

Apomorphine-induced rotation and cylinder test were assessed at the fifth week after 6-OHDA lesion before sacrifice in a double-blinded manner. For the apomorphine-induced rotation test, the rats received apomorphine 0.5 mg/kg (i.p.) and acclimated for 10 min. Data of asymmetry rotation were consecutively recorded for 30 min. For the cylinder test, the rats were placed in a glass cylinder with a height of 30 cm and diameter of 20 cm and the forelimb use was recorded for 5 min. Data of contralateral paw use was calculated as: (left paw use + bilateral paw use × 0.5)/(left paw use + right paw use + bilateral paw use) × 100%.

### Cell Culture and Treatment

Human neuroblastoma cells (SH-SY5Y) were purchased from the Cell Bank of the Institute of Biochemistry and Cell Biology (Shanghai, China). The cells were cultured in DMEM/F12 medium (Biological Industries) supplemented with 10% fetal bovine serum (Biological Industries) and 100 units/ml of penicillin/streptomycin. The cells were incubated at 37°C in a humidified atmosphere with 5% CO_2_.

### Cell Viability Assay

Cell viability was assessed with cell counting kit-8 (CCK-8, Dojindo Molecular Technologies, Japan) according toe the manufacture’s instruction. SH-SY5Y cells were seeded at a concentration of 10 × 10^3^ per well in a 96-well plate. After different treatments for 24 h, 10 μL of CCK-8 was added to each well and incubated at 37°C for 1 h. The absorbance was measured by a microplate reader at 450 nm.

### Adenovirus Infection

Adenovirus Ad-mCherry-GFP-LC3B was purchased from Beyotime (Beijing, China) to assess the state of autophagic flux *in vitro*. SH-SY5Y cells were seeded at a concentration of 10 × 10^4^ per well in a 6-well plate and infected with the adenovirus at a multiplicity of infection (MOI) of 10. The medium was changed 24 h after infection followed by corresponding treatments.

### Cell Transfection

For Atg7 and TFEB knockdown, siRNAs were synthesized by GenePharma (Shanghai, China) and applied. SH-SY5Y cells were seeded in 6-well plates at a density of 20 × 10^4^ per well, and 6 μL of jetPRIME reagent (Polyplus Transfection, France) was applied for siRNA transfection as instructed by the manufacture. The silencing efficiency was assessed with RT-PCR 24 h after transfection.

### Lysosome Labeling With LysoTracker Red

LysoTracker red (Beyotime) was applied to label lysosomes according to the manufacture’s instruction. In summary, at the end of corresponding treatment, SH-SY5Y cells seeded in 6-well plates were incubated with LysoTracker red diluted at a concentration of 1:15,000 (in DMEM/F12 medium) at 37°C for 30 min and replaced with fresh DMEM/F12 medium. The images were captured under the fluorescence microscope and further analyzed with ImageJ software as described before (Bonet-Ponce et al., 2016).

### Western Blot Analysis

Western blot was carried out with brain or whole cell extracts obtained by lysing with ripa buffer containing protease and phosphatase inhibitors, and protein concentration was determined with a BCA kit (Beyotime). Equal amount of protein samples were subjected to electrophoresis and then transferred to PVDF membranes (Millpoire, U.S.A.). The membranes were blocked in 5% non-fat milk in TBS with 0.1% Tween 20 (TBST) for 1 h at room temperature followed by incuabation of primary antibodies overnight. After washing the membranes with TBST for three times, they were further incubated with HRP-conjugated secondary antibodies for 2 h at room temperature and visualized with enhanced chemiluminescence (ELC) detection kit (Millipore). The results were quantified with ImageJ software and normalized with the loading control GAPDH.

### Real-Time Polymerase Chain Reaction

Total RNA was isolated from SH-SY5Y cells with RNAiso Plus (Takara) according to the manufacture’s instrunction and RNA quality and concentration were determined with spectrophotometer. Rt-PCR was performed with One-Step SYBR PrimeScript RT-PCR kit (Takara) in 7,500 Fast RT-PCR System (Applied Biosystems). The expression of GAPDH mRNA was used as an internal control. And the results were analyzed with–ΔΔCt method normalized with GAPDH.

### Immunofluorescence Staining

Sacrificed rats were perfused with normal saline followed by 4% paraformaldehyde. Then the whole brain was fixed in 4% paraformaldehyde overnight followed by dehydration with 30% sucrose for 2 days. Coronal brain sections containing midbrain were prepared at 15 μm with a freezing microtome (Leica, Germany). And SH-SY5Y cells were cultured on coverslips and after corresponding treatments, they were fixed with 4% paraformaldehyde in PBS for 15 min at room temperature. The fixed sections or cells were permeabilized with 0.1% Triton X-100 for 10 min at room temperature and blocked with 5% goat serum for 1 h at room temperature, followed by incubation of primary antibodies at 4°C overnight. After three times washing with PBS, they were incubated with Alexa Fluor 488 conjugated goat anti-mouse and Alexa Fluor 594 conjugated goat anti-rabbit secondary antibodies for 2 h at room temperature, and nuclei were stained with DAPI for 5 min at room temperature. All the images were acquired with a Nikon 300 microscope.

### Cell Apoptosis Assay

Cell apoptosis was detected with Annexin V-FITC/PI apoptosis assay kit (Bimake). After harvesting the SH-SY5Y cells with different treatments, they were resuspended in the staining buffer and stained with Annexin V-FITC and PI to assess cell death with flow cytometry (BD Bioscience). The apoptosis rate was calculated as “[Annexin V (+)PI(−) cells + Annexin V (+)PI(+) cells]/total cell × 100%”.

### Statistical Analysis

The data were presented as mean ± SD. And statistical analysis was carried out with Student’s t-test or one-way analysis of variance (ANOVA) followed by Bonferroni’s multiple group comparison using GraphPad Prism 6.0 Software (GraphPad Software Inc., U.S.A.) and SPSS 15.0 software (SPSS Science, U.S.A.). Statistical significance was accepted with *p* < 0.05.

## Results

### Ghrelin Could Improve Behavioral Test and Protect Dopaminergic Neurons Against 6-OHDA-induced Neurotoxicity by Activating Its Receptor

**FIGURE 1:**
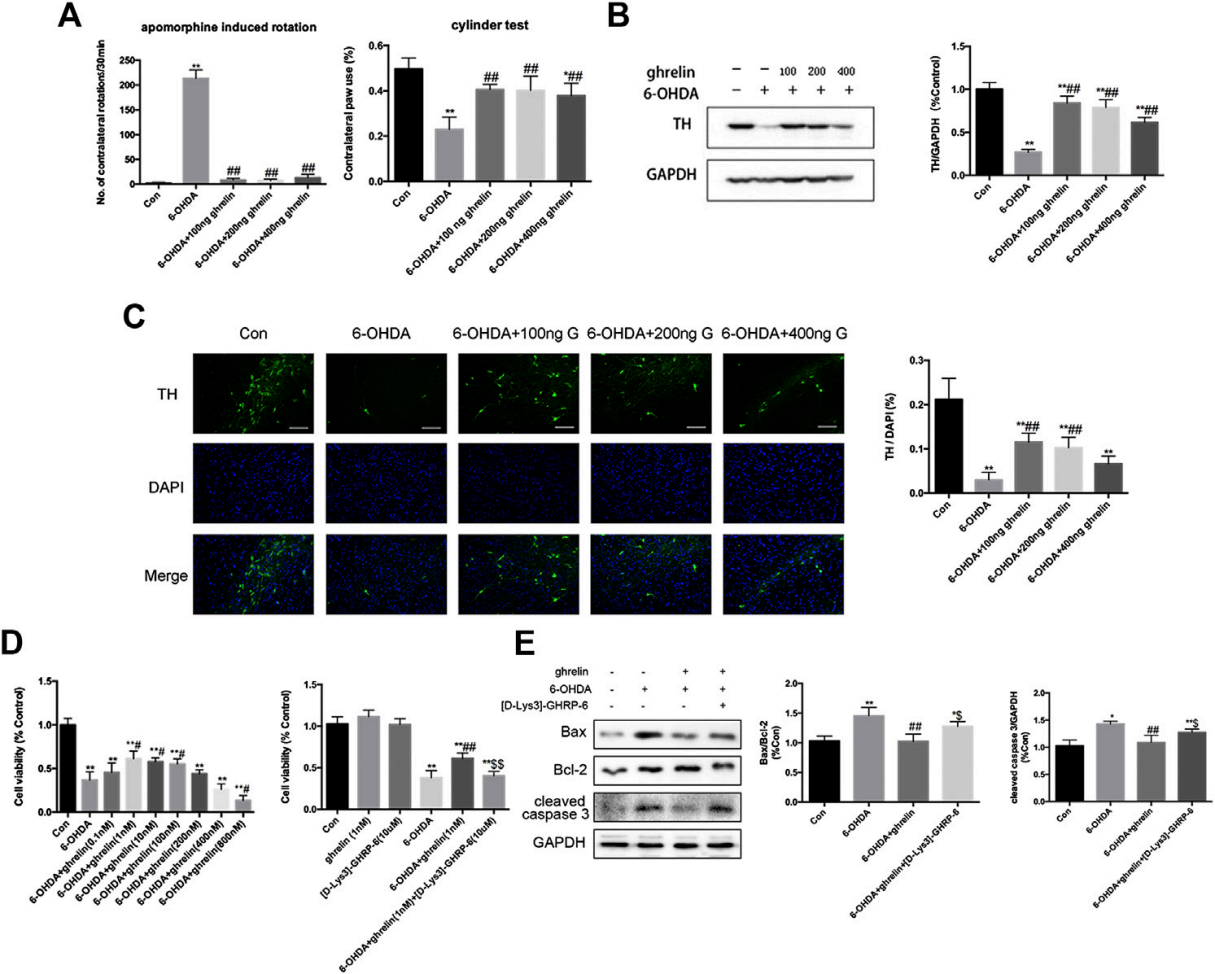
Ghrelin is protective against 6-OHDA-induced neurotoxicity. **(A)** The effect of ghrelin on apomorphine-induced rotations and cylinder test 5 weeks after 6-OHDA intoxication. The net number of apomorphine-induced rotations/30 min and the percentage of contralateral paw use were recorded. **(B)** Representative western blot and quantification of TH expression in the right substantia nigra 5 weeks after 6-OHDA intoxication; GAPDH was applied as the loading control and for band density normalization. **(C)** Representative immunofluorescence and quantification of TH n the right substantia nigra 5 weeks after 6-OHDA intoxication, scale bar = 50 μm. **(D)** Cell viability assessment of SH-SY5Y cells treated with 6-OHDA with different concentrations of ghrelin or 10 μM [D-Lys3]-GHRP-6 for 24 h. **(E)** Representative western blot and quantification of apoptosis-related markers expression in the right substantia nigra 1 day after 6-OHDA intoxication. Data are presented as mean ± SD; *p < 0.05 vs. the control group, **p < 0.01 vs. the control group, #*p* < 0.05 vs. the 6-OHDA group, ##*p* < 0.01 vs. the 6-OHDA group, $$*p* < 0.01 vs. the 6-OHDA + ghrelin group, n = 5.

To assess whether ghrelin could ameliorate 6-OHDA-induced motor dysfunction, apormorphine-induced rotation and cylinder test were assessed. As shown in Figure 1A, 6-OHDA induced a marked increase in the number of contralateral rotations, while different doses of ghrelin treatment (100 ng, 200 ng, 400 ng/day) could all effectively reduce apormorphine-induced contralateral rotations. Moreover, no statistical difference was observed in terms of the number of apormorphine-induced rotation among the groups with different doses of ghrelin treatment. And for the cylinder test, 6-OHDA could lead to a notable drop in the percentage of contralateral paw use, which could be partially relieved by different doses of ghrelin treatment (*p* < 0.01).

To study the effect of ghrelin on the survival of dopaminergic neurons, expression of tyrosine hydroxylase (TH) was assessed with western blot and immunofluorescence staining. As TH is the rate-limiting enzyme in catecholamine biosynthesis and decreased TH level is closely related to reduced dopamine synthesis, it is conventionally regarded as an important marker of dopaminergic neuron survival (Haavik and Toska, 1998). As shown in Figure 1B 6-OHDA induced a remarkable decrease in TH level in the rat substantia nigra 5 weeks after intrastriatal injection, which could be reversed by different doses of ghrelin treatment. However, unexpectedly, a dose-dependent decrease in TH expression was observed with increasing the dose of ghrelin treatment. Immunofluorescence staining of TH at the right substantia nigra (SN) also revealed a similar trend that 6-OHDA induced a dramatic loss of TH-positive neurons, which could be effectively reversed by 100 and 200 ng treatment of ghrelin (Figure 1C). Based on these findings, 100 ng ghrelin was applied for further experiments to study its regulation on autophagy and apoptosis *in vivo*. In SH-SY5Y cells, a similar trend could be observed, with 1 nM ghrelin treatment showing the best neuroprotective effect against 6-OHDA-induced neurotoxicity and 800 nM ghrelin treatment showing even deteriorating cell viability compared with 6-OHDA treatment alone (Figure 1D), and 1 nM ghrelin was applied for further experiments *in vitro*.

To further study whether the neuroprotective effect of ghrelin is dependent on its receptor, its receptor antagonist, [D-Lys3]-GHRP-6, was applied both *in vivo* and *in vitro*. As shown in Figure D, [D-Lys3]-GHRP-6 could abolish the neuroprotective effect of 1 nM ghrelin as revealed by a decline of cell viability. Moreover, by assessing the apoptosis-related markers 1 day after 6-OHDA treatment *in vivo*, [D-Lys3]-GHRP-6 could partially reverse the decline in bax/bcl-2 ratio as well as cleaved caspase three expression by ghrelin treatment (Figure 1E), suggesting that the neuroprotective effect of ghrelin is at least partially dependent on its receptor.

### Ghrelin Induces Autophagy Activation in 6-OHDA-induced Parkinson’s Disease Rat Model

**FIGURE 2:**
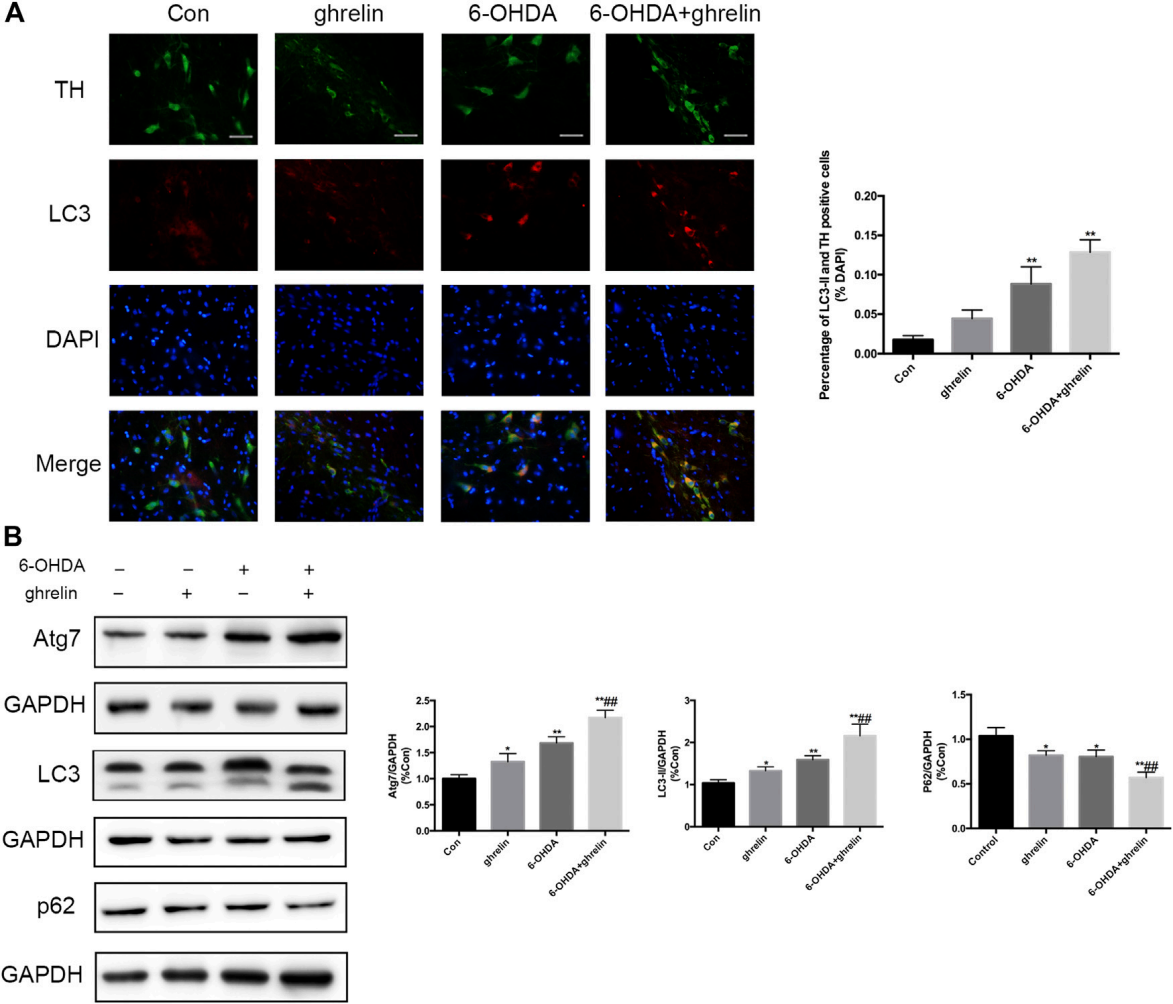
Ghrelin activates autophagy in 6-OHDA-induced PD rats. **(A)** Representative double immunofluorescence staining and quantification for LC3 and TH in the right substantia nigra 1 day after 6-OHDA treatment, scale bar = 50 μm. **(B)** Representative western blot and quantification of Atg7, LC3 and p62 in the right substantia nigra 1 day after 6-OHDA intoxication; GAPDH was applied as the loading control and for band density normalization. Data are presented as mean ± SD; *p < 0.05 vs. the control group, **p < 0.01 vs. the control group, ##*p* < 0.01 vs. the 6-OHDA group, n = 5.

To characterize the effect of ghrelin on autophagy in 6-OHDA-induced rat model, the rats were sacrificed 1 day after 6-OHDA treatment as our previous study suggested the most notable change at this time point (He et al., 2018). The expression of autophagy-related protein LC3 and dopaminergic neuron marker TH was assessed with double immunofluorescence staining (Figure 2A). An increased LC3 signal could be observed in the right SN of the rats treated with 6-OHDA or 6-OHDA + ghrelin compared with the control group, which colocalized with TH staining, suggesting an activation of autophagy in dopaminergic neurons. However, despite a light increase in the number of TH and LC3 double positive cells in the 6-OHDA + ghrelin, there was no statistical difference compared to that of the 6-OHDA group. As for the participation of the microtubule-associated protein LC3 in autophagosome biogenesis, LC3-II is modified by addition of phosphatidylethanolamine to LC3-I and serves as a maker for complete autophagosome formation (Klionsky et al., 2016), LC3-II, instead of LC3, is a better readout of the autophagy activation process. To further characterize the effect of ghrelin on autophagy activation, several autophagy markers including Atg7, LC3-II and p62 were assessed with western blot (Figure 2B). Atg7 is a noncanonical E1 enzyme that is involved in autophagosome formation (Hong et al., 2011), and p62 is one of the substrates in the autophagy process that can serve as a readout of autophagic degradation (Benito-Cuesta et al., 2017). As shown in Figure 2B, both 6-OHDA and ghrelin could induce a notable increase in Atg7 and LC3-II expression compared with the control group. And compared with the 6-OHDA treatment group, ghrelin treatment could further upregulate the expression of Atg7 and LC3-II. In contrast, compared with the control group, a marked decrease in p62 level could be observed in either single ghrelin or 6-OHDA group, and the expression of p62 was significantly lower in the ghrelin+6-OHDA group compared with the 6-OHDA group. Taken together, these results indicated that ghrelin could further induce autophagy activation in 6-OHDA-induced PD rat model.

### Ghrelin Modulates Autophagic Flux in 6-OHDA-induced SH-SY5Y Cell Model

**FIGURE 3:**
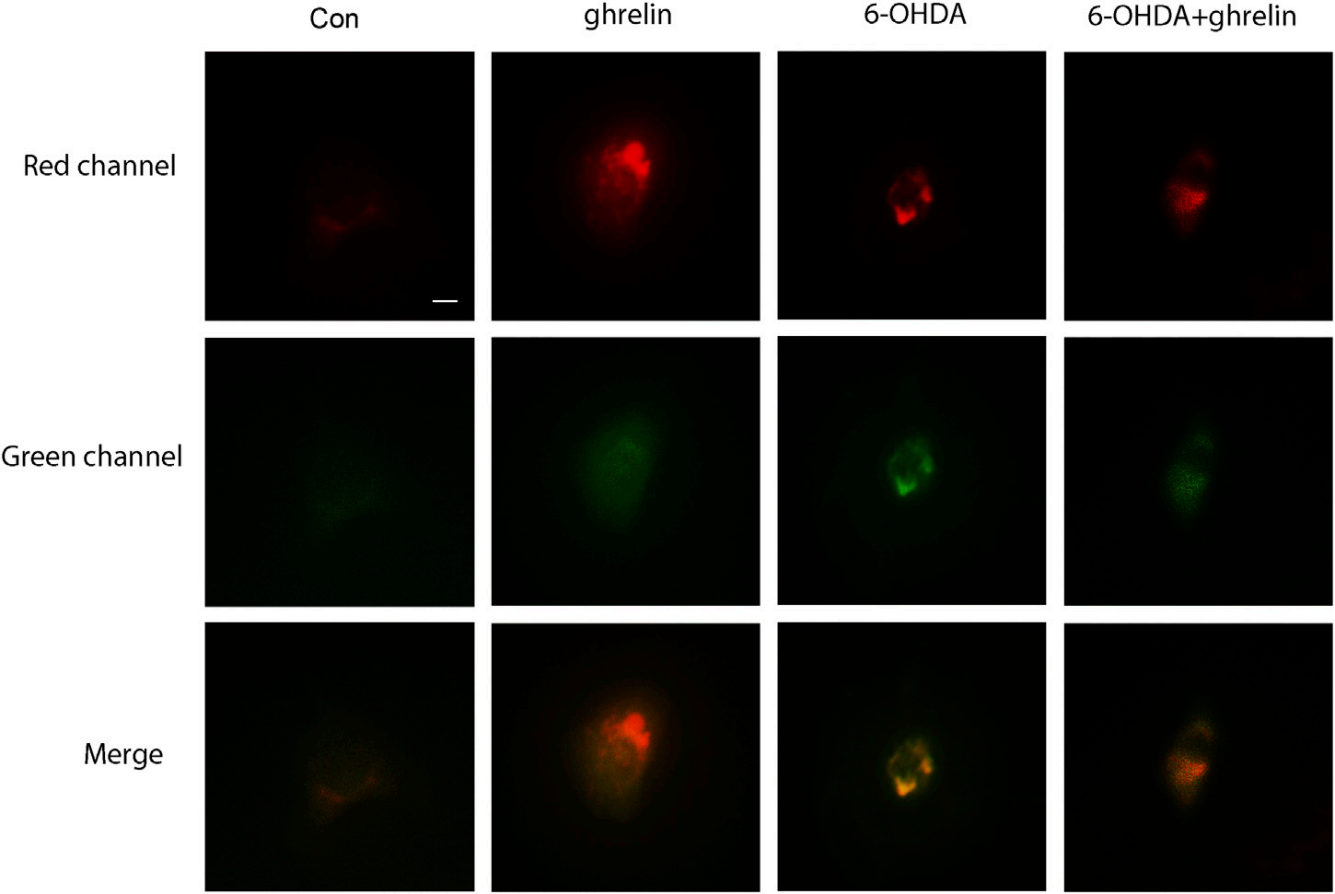
Ghrelin alleviates 6-OHDA-induced autophagic flux dysfunction. SH-SY5Y cells infected with Ad-mCherry-GFP-LC3 were treated with ghrelin, 6-OHDA or ghrelin and 6-OHDA for 24 h and the sate of autophagic flux was assessed under fluorescence microscope, scale bar = 10 μm.

As our previous study has revealed autophagic flux dysfunction in 6-OHDA-induced PD model, which contributed to the neuron death (He et al., 2018), and the *in vivo* results above could not fully reveal the actual state of autophagic flux upon ghrelin treatment, we further applied adenovirus Ad-mCherry-GFP-LC3B to assess the effect of ghrelin on autophagic flux in the 6-OHDA-induced SH-SY5Y cell model. We tinfected SH-SY5Y cells with Ad-mCherry-GFP-LC3B, further treated with ghrelin and 6-OHDA for 24 h and assessed the autophagic flux under the fluorescent microscope. Upon infection of Ad-mCherry-GFP-LC3B, autophagosomes are labeled with yellow signals due to both mCherry and GFP fluorescence; however, upon autophagosome and lysosome fusion, the autolysosomes will present red puncta as GFP signal is more rapidly quenched by the low PH of lysosomes. As shown in Figure 3, cells in the control group showed a weak and diffused signal in both green and red, while single ghrelin treatment could lead to a marked increase in red puncta, indicating an increased autophagosome and lysosome fusion. In contrast, 6-OHDA treatment alone yielded more yellow puncta, suggesting inadequate autophagosome and lysosome fusion. This could be partially reversed by ghrelin treatment, suggesting that ghrelin could promote more autophagosome and lysosome fusion and partially alleviate the autophagic flux dysfunction induced by 6-OHDA.

### The Anti-apoptosis Effect of Ghrelin Is Related to Autophagic Flux Regulation

**FIGURE 4:**
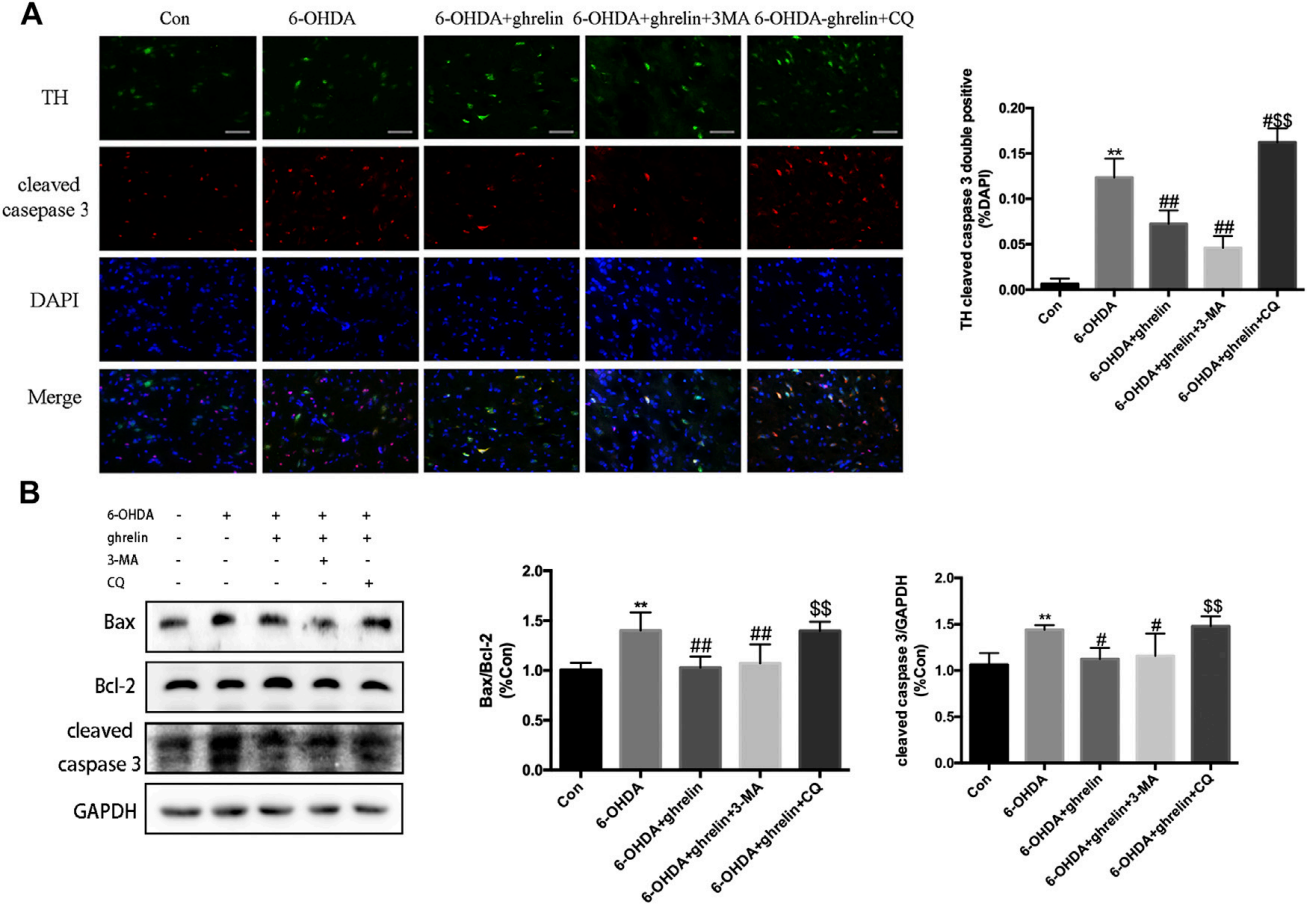
The anti-apoptosis effect of ghrelin is related to autophagic flux regulation. **(A)** Representative double immunofluorescence staining and quantification of cleaved caspase three and TH in the right substantia nigra 1 day after 6-OHDA treatment, scale bar = 50 μm. **(B)** Representative western blot and quantification of bax, bcl-2 and cleaved caspase three in the right SN 1 day after 6-OHDA intoxication; GAPDH was applied as the loading control and for band density normalization. Data are presented as mean ± SD; **p < 0.01 vs. the control group, #*p* < 0.05 vs. the 6-OHDA group, ##*p* < 0.01 vs. the 6-OHDA group, $$*p* < 0.01 vs. the 6-OHDA + ghrelin group, n = 5.

To elucidate whether the neuroprotective effect of ghrelin was due to autophagic flux regulation, two inhibitors that block different autophagy stages were applied. 3-methyladenine (3-MA) could inhibit class III phosphoinositide 3-kinase-dependent formation of autophagosomes, while chloroquine could effectively inhibit the fusion between autophagosomes and lysosomes. The doses and application of 3-MA and CQ have been validated in our previous study (He et al., 2018). As shown in Figure 4A, 6-OHDA induced a remarkable increase in the number of TH and cleaved caspase three double positive cells, which could be partially reversed by ghrelin treatment. Moreover, inhibiting autophagy induction with 3-MA showed a slight decrease in the number of TH and cleaved caspase three double positive cells compared with 6-OHDA + ghrelin group, but with no statistical difference. While chloroquine could induce a remarkable increase in the number of TH and cleaved caspase three double positive cells compared with 6-OHDA + ghrelin group Western blot of apoptosis-related markers also revealed a parallel trend. Ghrelin could notably reverse 6-OHDA-induced increase in bax/bcl-2 ratio as well as cleaved caspase three level, and 3-MA treatment did not further cause any remarkable change in bax/bcl-2 ratio and cleaved caspase three level compared with 6-OHDA + ghrelin group at 1 day post treatment. On the other hand, chloroquine induced a marked increase in bax/bcl-2 ratio and cleaved caspase three level compared with 6-OHDA + ghrelin group, suggesting that chloroquine treatment could abolish the neuroprotective effect of ghrelin (Figure 4B).

### Ghrelin Preserves TFEB Expression and Reverses 6-OHDA-induced Decrease in Lysosomes

**FIGURE 5:**
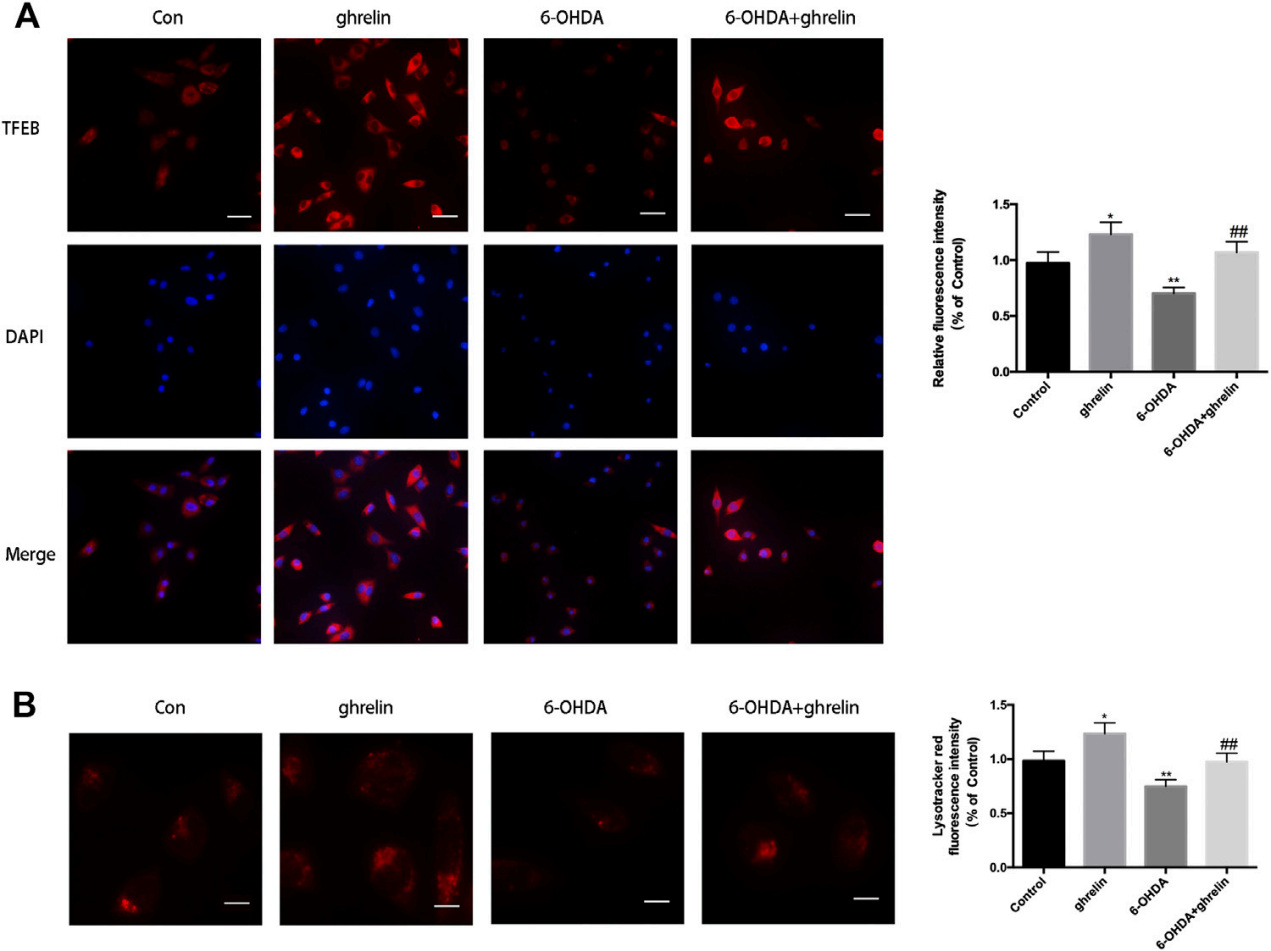
Ghrelin preserves lysosomes and TFEB expression against 6-OHDA-induced neurotoxicity. **(A)** Representative immunofluorescence staining for TFEB in SH-SY5Y cells treated with ghrelin, 6-OHDA or ghrelin and 6-OHDA for 24 h, scale bar = 50 μm. **(B)** Representative lysotracker staining and quantification of SH-SY5Y cells treated with ghrelin, 6-OHDA or ghrelin and 6-OHDA for 24 h, scale bar = 10 μm. Data are presented as mean ± SD; *p < 0.05 vs. the control group, **p < 0.01 vs. the control group, ##*p* < 0.01 vs. the 6-OHDA group, n = 5.

As autophagosomes requires constant fusion with lysosomes for further digestion and recycling, the state of lysosomes is critical to a healthy autophagic flux. Our previous study has revealed that 6-OHDA could impair the autophagic flux by downregulating TFEB (He et al., 2018), which is a critical transcription regulator for lysosome biosynthesis and function. We further assessed the effect of ghrelin on TFEB expression with immunofluorescence staining. As shown in Figure 5A, ghrelin could upregulate TFEB expression, while 6-OHDA induced a dramatic decline in TFEB fluorescent intensity, which could be partially reversed by ghrelin co-treatment. Moreover, ghrelin could not only induce a general increase in TFEB positive signal, an increase of TFEB positive signal in the nucleus compartment could also be observed, indicating an activation of the TFEB transcriptional activity (Figure 5A). To further validate the downstream effect of TFEB activation, lysosomes were visualized with LysoTracker staining in SH-SY5Y cells. As shown in Figure 5B, compared with control group, single ghrelin treatment could lead to a notable increase in LysoTracker red fluorescence intensity, while 6-OHDA treatment led to a drmatic decline (*p* < 0.01), which could be partially reversed by ghrelin treatment, further indicating that ghrelin could ameliorate 6-OHDA-induced autophagic flux dysfunction by preserving TFEB level and lysosome expression.

### Bidirectional Regulation of the Autophagic Flux Yields Opposite Effect on the Neuroprotective Action of Ghrelin

**FIGURE 6:**
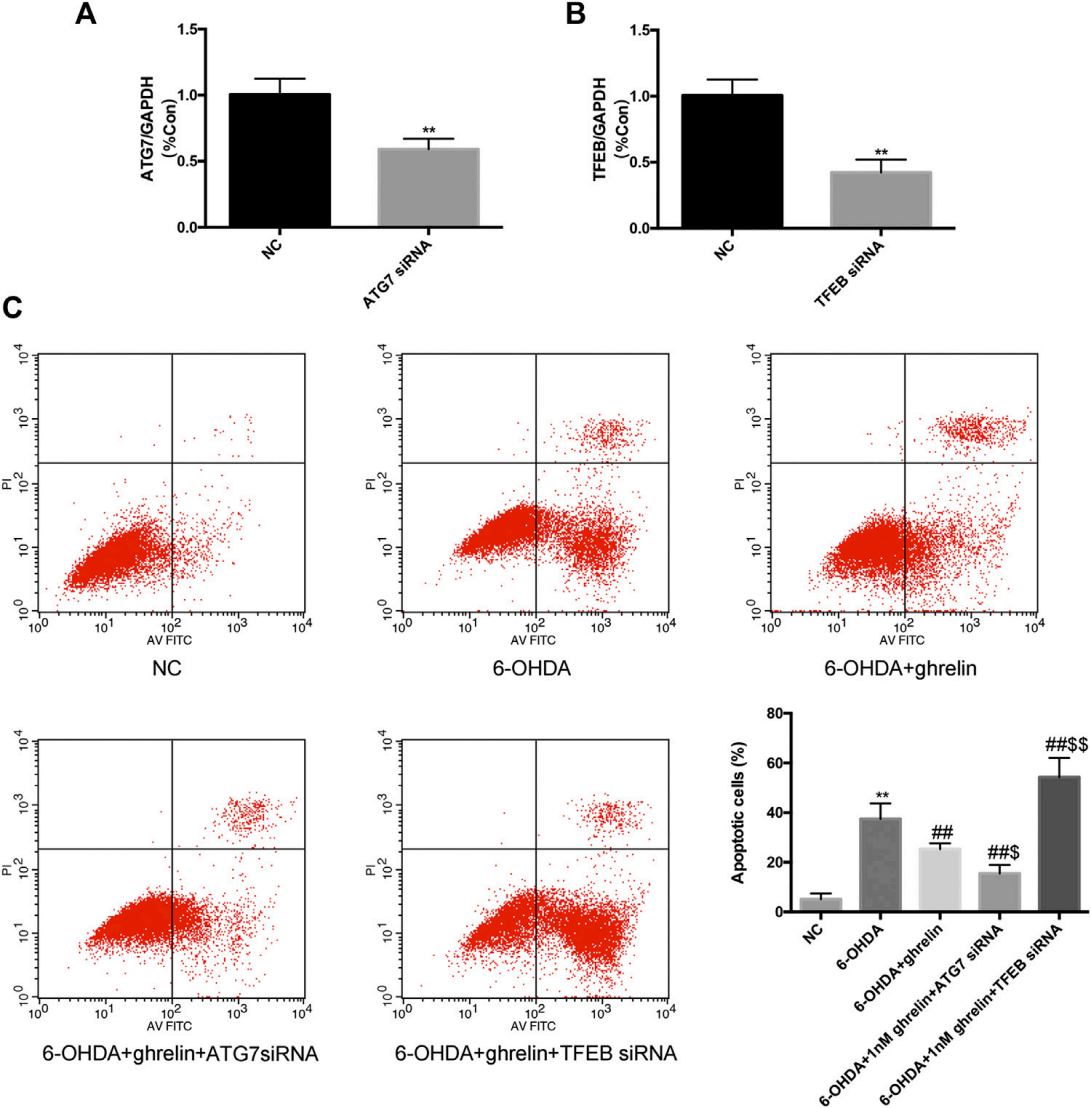
Downregulating Atg7 could boost while downregulating TFEB could abolish the anti-apoptotic effect of ghrelin against 6-OHDA-induced neurotoxicity. **(A)** RT-PCR analysis of ATG7 mRNA level in SH-SY5Y cells transfected with ATG7 siRNA; amplification of GAPDH cDNA was performed and served as internal control. **(B)** RT-PCR analysis of TFEB mRNA level in SH-SY5Y cells transfected with TFEB siRNA; amplification of GAPDH cDNA was performed and served as internal control. **(C)** Cell apoptosis was evaluated with Annexin V-PI/FITC kit through a flow cytometer. Data are presented as mean ± SD; **p < 0.01 vs. the NC group, ##*p* < 0.01 vs. the 6-OHDA group, $ *p* < 0.05 vs. the 6-OHDA + ghrelin group, $$*p* < 0.01 vs. the 6-OHDA + ghrelin group, n = 5.

To further validate the association between autophagic flux regulation and the neuroprotective action of ghrelin, we applied ATG7 siRNA and TFEB siRNA independently to inhibit autophagy activation and lysosome biogenesis and function. The silencing efficiency was validated with rt-PCR (Figure 6A,B). After transfecting SH-SY5Y cells with NC or siRNAs, they were further treated with 6-OHDA with or without ghrelin for 24 h. Cell apoptosis was assessed with Annexin V-PI/FITC kit through a flow cytometer. As shown in Figure 6C, 6-OHDA induced a notable increase in the number of apoptotic cells compared with the NC group, which could be partly reversed by ghrelin treatment. Moreover, ATG7 siRNA could further reduce the number of apoptotic cells compared with 6-OHDA + ghrelin treatment group (*p* < 0.05). In contrast, TFEB siRNA treatment showed an increase in the number of apoptotic cells, which was even higher than that in the 6-OHDA treatment group (*p* < 0.01). Collectively, these findings suggested that instead of via modulating autophagy activation, ghrelin exerts the neuroprotective effect against 6-OHDA more via preserving TFEB expression and thus relieving the autophagic flux dysfunction.

## Discussion

In the current study, we demonstrated that ghrelin is protective against 6-OHDA-induced neurotoxicity in SD rats and SH-SY5Y cells, which is closely related to improving the autophagic flux and promoting TFEB expression. As an endogenous neuropeptide, it has the advantage of reaching clinical settings faster compared to the synthesized compounds, and a full understanding of its underlying mechanism is important for clinical translation.

Early in 2008, Jiang et al. revealed that ghrelin could antagonize MPTP-induced neurotoxicity to the dopaminergic neurons in the substantia nigra (Jiang et al., 2008). Later their clinical study revealed that both the total ghrelin level and acylated ghrelin level are lower in PD patients than these in the normal controls. In addition, the postprandial ghrelin suppression effect and preprandial peak response are also impaired in PD patients (Song et al., 2017). Endogenous ghrelin has been demonstrated to mediate the neuroprotective effects of calorie restriction in MPTP-induced PD model (Bayliss et al., 2016b). And an *in vivo* study found that either pharmacological or genetic inhibition of endogenous ghrelin receptor could induce dopaminergic neuron dysfunction and extrapyramidal symptoms of PD (Suda et al., 2018). Moreover, exogenous acylated ghrelin, rather than unacylated ghrelin, is neuroprotective in MPTP-induced PD mouse model by inhibiting neuroinflammation (Bayliss et al., 2016a). So far, the neuroprotective actions of ghrelin mainly involve inhibiting microglial activation and neuroinflammation, antioxidation, improving mitochondrial redox state and antagonizing apoptosis (Andrews et al., 2009; Dong et al., 2009; Moon et al., 2009; Liu et al., 2010; Yu et al., 2016). Zane et al. reported that the neuroprotective effect of ghrelin was dependent on the mitochondrial redox state by UCP2-dependent alterations in the mitochondrial respiration, mitochondrial biogenesis, and ROS production (Andrews et al., 2009). In addition, in rotenone-induced PD model, Yu et al. found that ghrelin could reverse rotenone-induced reduced mitochondrial membrane potential, decreased activity of mitochondrial complex I and cytochrome C release (Yu et al., 2016). Herein, we identified a novel regulatory role of ghrelin on the state of autophagic flux in 6-OHDA-induced PD models, which further added evidence for ghrelin as a promising target for PD treatment.

As a highly conserved and dynamic cellular process that removes damaged organelles or proteins, autophagy could be divided into five stages: autophagosome induction, autophagosome elongation, autophagosome maturation, fusion with lysosome and degradation. Despite that the effect of ghrelin on autophagy has been extensively studied, the results remain controversial. One recent study revealed that ghrelin could induce autophagy via SIRT1/AMPK axis in lymphoblastic leukemia cell lines (Heshmati et al., 2020), and Xu et al. also revealed that ghrelin could vascular autophagy in the rats with vascular calcification (Xu et al., 2017). While Chung et al. reported that ghrelin could protect adult rat hippocampal neural stem cells from excessive autophagy during oxygen-glucose deprivation (Chung et al., 2018), and Wang et al. revealed that ghrelin could protect against DOX-induced cardiomyocyte apoptosis via autophagy inhibition (Wang et al., 2014). Such discrepancy might be due to the different stimulus in varied disease models, the doses of ghrelin applied and varied tissue specificity. Herein, we identified an autophagy activation role of ghrelin as both Atg7 and LC3II levels were increased and more red puncta could be observed in SH-SY5Y cells transfected with Ad-mCherry-GFP-LC3 upon single ghrelin treatment. However, when blocking autophagy induction with 3-MA *in vivo* and ATG7 siRNA *in vitro*, we observed inconsistent results on the neuroprotective effect of ghrelin. Application of 3-MA showed no significant effect on the expression of apoptosis-related makers compared with 6-OHDA + ghrelin group, while ATG7 siRNA could further reduce the number of apoptotic cells compared with 6-OHDA + ghrelin treatment. Such discrepancy could be explained by the fact that the *in vivo* study was carried out at only 1 day after different treatments. Despite our previous study has revealed a most notable change in autophagy related markers 1 day after 6-OHDA treatment *in vivo* (He et al., 2018), it would be too early to reveal the effect of 3-MA on apoptosis at that time point. While 6-OHDA treatment for 24 h *in vitro* would yield a stable cell loss around 50%, which could better assess the outcomes of different interventions. Our previous study found that 6-OHDA could lead to an instant activation of autophagy and cause a late-stage autophagic flux dysfunction both *in vivo* and *in vitro*, and either inhibiting autophagy activation or enhancing lysosome function could well relieve the autophagic flux dysfunction and protect against 6-OHDA-induced neurotoxicity (He et al., 2018), which was consistent with the *in vitro* result herein. Taken together, these results suggest that despite that ghrelin could promote autophagy activation, it is not the fundamental mechanism to confer its neuroprotective effect. Also it’s notable that one *in vivo* study based on Goat (−/−) mice model revealed reduced autophagy in the livers of fasted, fat-depleted mice, suggesting an autophagy inducing role of endogenous ghrelin (Zhang et al., 2015). It would be intriguing to investigate whether and how endogenous ghrelin modulates autophagy in different PD models with genetic modifying approaches.

Lysosomes are lytic and degradative organelles that contain over 60 soluble lysosomal hydrolases and play an important role in maintaining intracellular homeostasis. Mounting evidence suggests a close link between lysosome abnormality and PD. For instance, one of the lysosomal gene GBA1, that encodes lysosomal β-glucocerebrosidase is one of the most common genetic risk factor of PD (Sidransky et al., 2009). In addition, postmortem PD brains also showed a remarkable decrease in lysosomal markers compared to the control brains (Dehay et al., 2010). With regard to 6-OHDA-induced PD models, our previous study revealed a decrease in both lysosome expression and function, and we found that instead of focusing on autophagy induction, restoration of lysosome function to relieve the autophagic flux burden could well convey a neuroprotective effect (He et al., 2018). In the current study, we observed that ghrelin could improve lysosome expression via upregulating TFEB expression, and the protective effect of ghrelin against 6-OHDA-induced neurotoxicity was abolished by knockdown of TFEB or inhibiting autophagosome and lysosome fusion with chloroquine. One study by Witek et al. revealed that exogenous ghrelin treatment could promote lysosome function via inducing an increase in the activity of a number of lysosomal enzymes, including cathepsin D, cathepsin L, alanine aminopeptidase, lysosomal lipase, etc.(Witek et al., 2005). It could partially explain our finding that high concentration of ghrelin exacerbated neuronal death instead of yielding a further neuroprotective effect due to excessive lysosomal activity or excessive autophagy. Collectively, these findings suggest that compared with enhancing autophagy induction, improving autophagic flux dysfunction via restoring TFEB level and promoting autophagosome and lysosome fusion is more critical to the neuroprotective action of ghrelin.

One interesting finding in the current study is that we observed a decline in the neuroprotective effect of ghrelin with increasing its dose *in vivo* or concentration *in vitro*. Herein, we observed a loss of neuroprotection for ghrelin exceeding 200 ng *in vivo* or 200 nmol *in vitro*. While Gong et al. reported an increase in ghrelin’s ability to promote midbrain neural stem cell differentiation with increasing its concentration up to 1 μM (Gong et al., 2020). Moreover, Wang et al. reported that 10 μM ghrelin could confer equal protection in SH-SY5Y cells against MPP^+^ neurotoxicity as 1 μM ghrelin (Wang et al., 2020). Despite that we observed a decline in cell viability with co-treatment of 6-OHDA and ghrelin over 200 nmol, one single treatment of ghrelin over 200 nmol showed no obvious effect on cell viability in SH-SY5Y cells (data not shown), suggesting that a high concentration of ghrelin alone would not be the reason of loss of cell viability. We speculate that the effect of ghrelin at high concentration/dose would vary depending on the cell or animal model studied. In the current 6-OHDA-induced PD models, a loss of neuroprotection at high ghrelin concentrations would be attributed to the over activation of autophagy and exacerbated autophagic flux dysfunction.

There are several limitations in the current study. First, we only assessed the autophagy related changes *in vivo* at day 1 after treatment as this point showed the most notable change in 6-OHDA-induced rat model according to our previous study (He et al., 2018). As autophagy is a highly dynamic process, more time points or application of genetic modifying techniques to characterize the state of autophagic flux *in vivo* could better reveal the modulation of ghrelin on autophagy. In addition, the effect of ghrelin has been revealed to be cell type dependent (Cecarini et al., 2016). In the current study, we only applied SH-SY5Y cell line, which is one of the most common cell lines for PD modeling. Further studies on primary midbrain neurons would be necessary. Moreover, we only studied the effect of exogenous ghrelin in 6-OHDA induced PD models. As ghrelin is an endogenous neuropeptide, a thorough study on the function of endogenous ghrelin in PD would also be crucial.

## Conclusion

Ghrelin is protective agianst 6-OHDA-induced neurotoxicity in SD rats as well as SH-SY5Y cells. The underlying mechanism involves inhibition of apoptosis and regulation of autophagic flux. Despite an action of autophagy activation, the neuroprotective effect of ghrelin is more reliant on restoration of TFEB level and relief of autophagic flux dysfunction. This study suggests the importance of preserving functional autophagic flux against neurodegeneration and provides further basis for ghrelin as a potential target for PD.

## Data Availability Statement

The raw data supporting the conclusions of this article will be made available by the authors, without undue reservation.

## Ethics Statement

The animal study was reviewed and approved by Shengjing Hospital Medical Ethics Committee.

## Author Contributions

JF and YG designed experiments, reviewed and edited the manuscript. XH, WY and FL carried out cell and rat experiments. XH and WY analyzed the data and wrote the manuscript. All the authors read and approved the manuscript.

## Funding

This study was supported by the National Natural Science Foundation of China (Grant No. 81901417; 2019), the 30s project of Shengjing Hospital (Grant No. M0292), the Natural Science Foundation Doctoral Research Initiation Plan of Liaoning Province (Grant No. 2019-BS-287; 2019), the China Postdoctoral Science Foundation (Grant No. 2019M661173; 2019), the Faculty Research Support Plan of China Medical University (Grant No. QGZD2018069).

## Conflict of Interest

The authors declare that the research was conducted in the absence of any commercial or financial relationships that could be construed as a potential conflict of interest.
